# Autism-Related Differences in Cortical Activation When Observing, Producing, and Imitating Communicative Gestures: An fNIRS Study

**DOI:** 10.3390/brainsci13091284

**Published:** 2023-09-04

**Authors:** Wan-Chun Su, McKenzie Culotta, Jessica Mueller, Daisuke Tsuzuki, Anjana N. Bhat

**Affiliations:** 1 Department of Physical Therapy, University of Delaware, Newark, DE 19713, USA; wcsu@udel.edu (W.-C.S.); mckenzieculotta@gmail.com (M.C.); 2 Biomechanics and Movement Science Program, College of Health Sciences, University of Delaware, Newark, DE 19713, USA; 3 Department of Behavioral Health, Swank Autism Center, Nemours Children’s Hospital, Wilmington, DE 19803, USA; jka.mueller@gmail.com; 4 Department of Information Sciences, Kochi University, Kochi 780-8520, Japan; tsuzukid@is.kochi-u.ac.jp; 5 Interdisciplinary Neuroscience Graduate Program, Department of Psychological and Brain Sciences, University of Delaware, Newark, DE 19713, USA

**Keywords:** autism spectrum disorder, functional near-infrared spectroscopy, communicative gesture, gesture production, gesture processing, imitation

## Abstract

Children with autism spectrum disorder (ASD) have difficulties in gestural communication during social interactions. However, the neural mechanisms involved in naturalistic gestural communication remain poorly understood. In this study, cortical activation patterns associated with gestural communication were examined in thirty-two children with and without ASD (mean age: 11.0 years, SE: 0.6 years). Functional near-infrared spectroscopy (fNIRS) was used to record cortical activation while children produced, observed, or imitated communicative gestures. Children with ASD demonstrated more spatial and temporal errors when performing and imitating communicative gestures. Although both typically developing (TD) children and children with ASD showed left-lateralized cortical activation during gesture production, children with ASD showed hyperactivation in the middle/inferior frontal gyrus (MIFG) during observation and imitation, and hypoactivation in the middle/superior temporal gyrus (MSTG) during gesture production compared to their TD peers. More importantly, children with ASD exhibited greater MSTG activation during imitation than during gesture production, suggesting that imitation could be an effective intervention strategy to engage cortical regions crucial for processing and producing gestures. Our study provides valuable insights into the neural mechanisms underlying gestural communication difficulties in ASD, while also identifying potential neurobiomarkers that could serve as objective measures for evaluating intervention effectiveness in children with ASD.

## 1. Introduction

Children with autism spectrum disorder (ASD) commonly exhibit impairments in social communication, encompassing both verbal and nonverbal/gestural communication aspects (DSM-5) [1]. These impairments manifest in various ways, including difficulties in recognizing communicative gestures [2,3], displaying atypical or fewer spontaneous gestures during interactions with caregivers [4,5], and experiencing delays in acquiring new gestures through imitation [6]. These difficulties in perceiving, producing, or imitating communicative gestures can result in miscommunication during social interactions and can negatively affect children’s relationships with peers and caregivers. Moreover, communicative gestures serve as a foundation for later language development, as caregivers frequently respond verbally to their infant’s gestures [7,8]. A limited repertoire of gestures can hinder infants’ opportunities to learn from verbal responses provided by their caregivers [7,8]. Considering the pivotal role of communicative gestures in social interaction and their profound impact on language development, investigating the underlying neural mechanisms provides a valuable framework for understanding social communication difficulties observed in children with ASD.

Functional magnetic resonance imaging (fMRI) studies have provided insights into the differences in neural activity related to ASD. For instance, when observing communicative gestures and functional pantomime actions, children with ASD exhibited greater activation over the inferior frontal gyrus (IFG) and reduced activation over the posterior superior temporal sulcus (STS) [9]. Similarly, during the performance of communicative gestures depicted on picture cards, children with ASD showed reduced activation in the angular gyrus and precentral gyrus, as well as the middle cingulate gyrus [10]. However, these studies have been limited by the constraints of fMRI, which restrict observations to two-dimensional videos or hand actions depicted on picture cards, without the inclusion of real-world, face-to-face interactions with others [9,10]. Research has indicated reduced corticospinal excitability when watching video recordings compared to observing live dance, suggesting that the videos may not elicit similar levels of cortical activation as naturalistic social interactions [11]. To address these limitations, the current study utilized functional near-infrared spectroscopy (fNIRS) to investigate cortical activation patterns associated with the observation, production, and imitation of communicative gestures during face-to-face interactions in children with and without ASD. Through this innovative approach, the current study aimed to identify neurobiomarkers that shed light on the neural mechanisms underlying the gestural difficulties of children with ASD. Moreover, these neurobiomarkers have the potential to serve as objective measures for evaluating the effectiveness of gesture-based interventions that are often used to improve communication skills of children with ASD [12,13].

**Figure 1:**
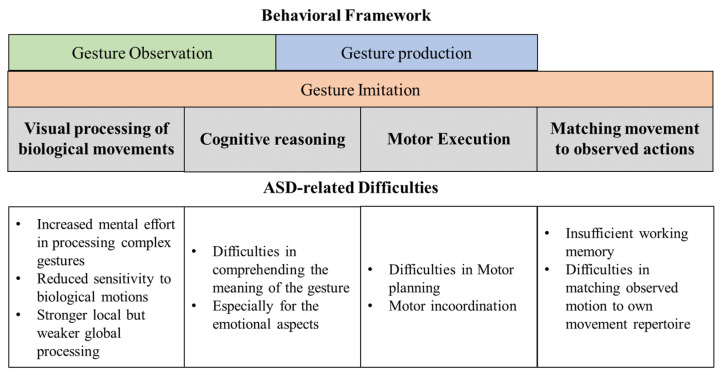
Behavioral framework and ASD-related difficulties in gesture observation, production, and imitation.

Non-verbal gestural communication relies on a complex interplay of sensorimotor and cognitive processes to perceive biological motions and comprehend (reasoning) the underlying meaning of the gestural bid [14,15]. In a study involving the observation of point light displays featuring two agents, a superior ability to detect and discriminate biological motions was observed during the communicative compared to the non-communicative condition, emphasizing the importance of reasoning in gesture perception [14]. Moreover, when imitating other’s gestures, it is suggested that reasoning and causal understanding are essential, in addition to working/episodic memory, as they contribute to the maintenance of the learned gesture over an extended period of time [15]. Please refer to Figure 1 for a behavioral framework for gesture observation, production, and imitation.

Neuroimaging studies show a highly left-lateralized fronto-temporo-parietal activation pattern while observing, producing, and imitating communicative gestures [16]. It is suggested that even during social interaction, some level of reasoning and physical judgment is present, as evidenced by overlapping fMRI-related activation over the frontal and parietal lobes when individuals observe physical and social interactions [17]. The right IPL is primarily associated with visuospatial processing, whereas the left IPL is involved in technical reasoning, a cognitive ability that helps individuals understand the causal effect of the physical world [18]. This form of reasoning encompasses both causal reasoning, allowing for predictions of action effects on the environment, and analogical reasoning, which enables the transfer of physical knowledge from one situation to another [16]. Despite the overall network, cortical activation varies during observation, production, and imitation of communicative gestures. Gesture observation predominantly activates frontal and parietal regions which are involved in processing visual and contextual information, whereas gesture production engages frontal and motor/supplementary motor areas to generate actions [19]. Gestural imitation, on the other hand, involves the observation–execution matching system (OEMS, encompassing the IFG, STS, and inferior parietal lobe (IPL), which is crucial for matching one’s own movements to those of others) [20,21]. 

More specifically, for gesture observation, a meta-analysis of fMRI studies revealed activation over IFG, precentral gyrus, IPL, middle and superior temporal gyrus (MSTG), and inferior temporal gyrus, as well as the precuneus, amygdala, and para-hippocampal gyrus during gesture observation [22]. Notably, observation of communicative gestures elicits additional cortical activation over the left ventrolateral prefrontal cortex compared to body-referencing gestures [23]. Moreover, STS is known to be sensitive to encoding the emotional aspects of observed gestures [23]. Neural networks involved in gesture production overlap with those important for speech generation. An fMRI study found activation over Broca’s area when the participants used speech or gestures to describe an object, suggesting its role in processing semantic aspects of gestures [24]. Similarly, strong overlapping activation in the parietal regions was observed when gestures and drawing were employed to describe an object, highlighting the importance of the parietal cortex in conveying information through movements [24]. Furthermore, Marstaller et al. (2015) found overlapping activation in the fronto-temporal regions, including the IFG, STS, primary motor, and premotor areas, as well as the hippocampus and para-hippocampus regions during speech and gesture production [25]. Regarding gestural imitation, the OEMS, including the connections between the IFG, STS, and IPL regions, plays a crucial role [20,21]. The STS region is involved in establishing visuomotor correspondence and actively matching one’s own movements with observed actions [26]. The IFG region plays a role in perceiving the salience of action information and inferring the intentions of social partners [27], while the IPL region is involved in anticipating and planning the movement kinematics necessary to match one’s own movements with those of others [28]. Compared to transitive gestures, the imitation of communicative gestures may require greater temporal activation, as it is important for semantic processing [29]. Taken together, fronto-temporo-parietal cortical networks, especially the IFG, STS, and IPL regions, are important for observing, producing, and imitating communicative gestures.

Children with ASD exhibit difficulties in accurately perceiving and recognizing communicative gestures [2,3,4]. A study measuring pupillary dilation during communicative gesture observation suggested that children with ASD exerted increased mental effort (reflected by reduced pupillary dilation), particularly when perceiving more complex gestures compared to neurotypical controls [30]. These challenges in perceiving communicative gestures may stem from autistic children’s difficulties in processing and interpreting biological motions. Children with ASD were found to have reduced sensitivity and a diminished preference when observing biological motions [31,32], probably due to their atypical visuospatial-processing performance (e.g., stronger local but weaker global processing, better discrimination between targets and distractors) (Figure 1) [33,34,35,36]. Additionally, they have difficulties comprehending the emotional nuances and underlying intentions or goals behind others’ actions [37,38]. Atypical activation patterns have been reported in individuals with ASD when viewing gestures, particularly in the medial frontal gyrus, IFG, STS, premotor cortex, and amygdala [9,37,39]. Prefrontal regions, including IFG, play a crucial role in understanding the semantic and emotional aspects of communicative gestures [22,40,41], while STS is involved in observing biological motions and matching observed actions to one’s own movement repertoire [26,42]. The atypical activation observed in the prefrontal cortex and STS regions activation may reflect the difficulties experienced by children with ASD in interpreting the intentions, emotions, and biological hand motions conveyed through observed gestures.

The production and imitation of communicative gestures in individuals with ASD also deviate from typical patterns across lifespan. Toddlers with ASD use atypical or fewer spontaneous gestures during structured play with their caregivers [5]. A study in children at higher risk for ASD found that the quality of gesture production at 12 months predicted receptive language and ASD symptoms at 24 months [43]. Even adults with ASD exhibited atypical gestural form and function during conversations [44]. Additionally, even in deaf children with ASD who have practiced fingerspelling, more praxis errors and poorer receptive language abilities were observed compared to deaf children without ASD [3]. These challenges in gestural production and imitation might stem from their difficulties in motor planning or developmental dyspraxia [45,46]. For example, children with ASD often exhibit poor motor planning and coordination, which can impact their ability to imitate and synchronize their movements with social partners (Figure 1) [47,48]. In an fMRI study, children with ASD showed typical activation over premotor, IFG, IPL, insula, thalamus, and occipital regions when imitating communicative gestures shown on picture cards [10]. However, atypical activation was observed in the angular gyrus, precentral gyrus, and middle cingulate gyrus when imitating communicative gestures performed by others [10]. To date, no study has reported ASD-related differences in cortical activation during the performance of communicative gestures during a naturalistic, face-to-face interaction with social partners.

With the recent progress in neuroimaging techniques, new tools have emerged that effectively account for motion artifacts and capture cortical activation during naturalistic social interactions in an upright position [49]. Using fNIRS, our research group has conducted studies on cortical activation in children with and without ASD during tasks involving imitation/synchronous reaching and whole-body sway with social partners [50,51,52,53]. Consistently, we have reported hypoactivation in the IFG and STS regions, along with hyperactivation in the IPL regions among children with ASD during imitation/synchronous limb and whole-body actions [50,51,52,53]. Additionally, we observed hyperactivation in the IPL and hypoactivation of the IFG and STS in children with ASD when performing tool-related gestures [54]. Although these fNIRS studies have suggested potential neurobiomarkers for children with ASD, none of them have specifically targeted communicative gestures. Considering the important role of communicative gestures in social interactions and the considerable challenges faced by children with ASD in this domain, there is an urgent need for fNIRS studies to examine ASD-related neural activity during gesture observation, production, and imitation. 

In short, previous fMRI studies have suggested potential mechanisms underlying ASD-related difficulties in gestural communication, however, these studies have been limited to observation of videos and pictures. It remains unclear if the findings from fMRI studies could be extended to real-world, face-to-face gestural communication. fNIRS appears to offer greater ecological validity, however, no studies have yet used fNIRS to investigate cortical activation during observation, production, and imitation of communicative gestures in children with and without ASD. Furthermore, the relationships between cortical activation, gestural performance, adaptive functioning, and ASD symptoms have not been explored. The current study aims to bridge these knowledge gaps by (1) investigating fNIRS-related cortical activation in children with and without ASD during real-time, communicative gesture observation, production, and imitation; and (2) investigating the correlations between gestural performance, adaptive functioning, and ASD symptoms, and cortical activation during gestural communication. We hypothesize that children with ASD will exhibit increased activation over the prefrontal regions when observing communicative gestures from social partners, alongside atypical fronto-temporal activation when producing and imitating communicative gestures in the context of social interactions. Moreover, we anticipate that these atypical fNIRS activation patterns will correlate with ASD symptoms, praxis performance, and adaptive functioning in children with ASD.

## 2. Materials and Methods

### 2.1. Participants

Thirty-two children, including those with and without ASD, participated in the study (mean age ± SE: ASD group: 11.1 ± 0.9, 11 males and 4 females; TD group: 10.8 ± 0.7, 11 males and 6 females, Table 1; *p*s > 0.05). Recruitment efforts involved posting online announcements, making phone calls, and distributing fliers through ASD advocacy groups, local schools, autism services, and community centers. Prior to enrollment, we conducted a phone interview with the parent to confirm their child’s eligibility and collect demographic information (i.e., age, sex, race/ethnicity, developmental history, etc.). Specifically, TD children were included if they were aged between 6 and 17 years old and excluded if they had any neurological or developmental diagnoses/delays, preterm birth, significant birth history, or family history of ASD. For children with ASD, the inclusion criteria were: (1) an age range of 6 to 17 years, along with (2) a professionally confirmed ASD diagnosis supported by a school record (e.g., Individualized Education Plan), and/or a medical or neuropsychological record from a psychiatrist or clinical psychologist (using the Autism Diagnostic Observation Schedule (ADOS) or Autism Diagnostic Interview-Revised (ADI-R) measures). Children with ASD were excluded if they were (1) unable to follow one-step instructions (e.g., “copy me” or “move on your own”), or (2) had significant sensory and behavioral issues that prevented them from wearing the fNIRS cap and engaging in the required communication tasks. In addition to the screening interview, a clinical psychologist (i.e., 3rd author) independently confirmed the diagnosis of ASD using the ADOS (average score ± SE = 18.9 ± 1.9) [55]. The level of intelligence in children with and without ASD was assessed using the Stanford–Binet IQ test by the same clinical psychologist/third author (Full Scale IQ ± SE: ASD: 82.1 ± 7.6; TD: 114.2 ± 1.7, *p* < 0.001) [56]. Additionally, we used the Social Communication Questionnaire (SCQ; averaged score ± SE: 24.5 ± 3.2) [57] to screen for ASD-related social communication symptoms, the Hollingshead Four-Factor Index of Socioeconomic Status (SES-Child) to estimate the socioeconomic status (averaged score ± SE: ASD: 65.9 ± 5.1, TD: 69.7 ± 4.4; *p* > 0.05), and the Coren’s handedness survey to determine their handedness (averaged score ± SE: ASD: 33.5 ± 1.5, TD: 33.4 ± 1.8; *p* > 0.05) [58]. Parents were requested to complete various questionnaires, including Vineland Adaptive Behavioral Scales—2nd edition (VABS) [59], Social Responsive Scale (SRS) [60], and Interpersonal Communication Scales (ICS; Table 1) [61]. All parents and children signed the consent/assent forms approved by the University of Delaware Institutional Review Board before study participation.

### 2.2. Experimental Procedures

**Figure 2:**
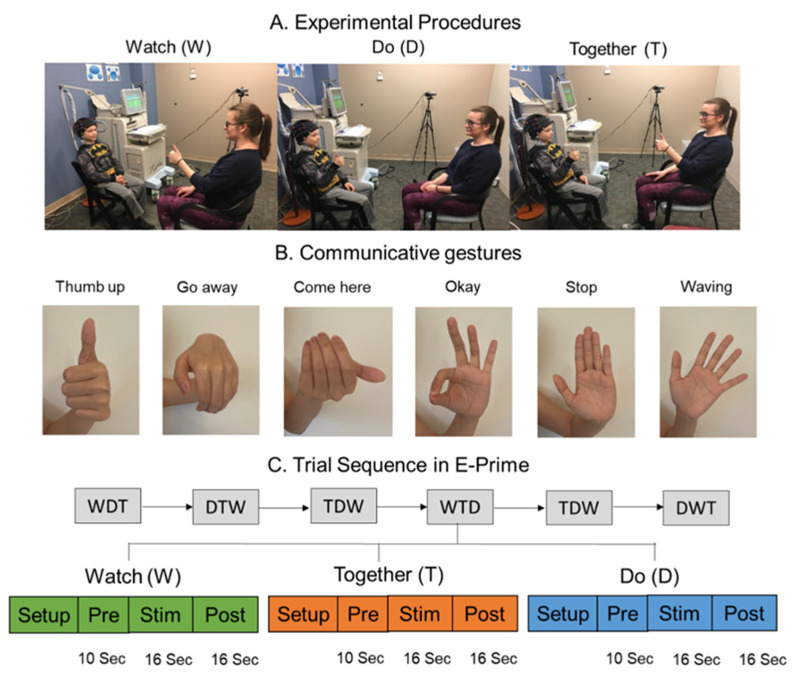
Experimental setup (**A**), communicative gestures (**B**), and task sequence (**C**) during the communicative gesture tasks. Written permission for publication of participant pictures has been taken.

During the fNIRS experiment, each participating child sat face-to-face with an adult tester wearing an fNIRS cap embedded with two 3 × 3 probe sets positioned on their head. The child completed communicative gesture tasks across three conditions: (1) Watch: the child observed the adult tester repeatedly performing the communicative hand gestures towards them; (2) Do: the child was shown a picture card depicting hand gestures and asked to perform the corresponding gestures towards the adult tester repeatedly; and (3) Together: the child was instructed to copy and synchronize their gestures with those demonstrated by the adult (Figure 2A). Six common communicative gestures were used in this experiment, including thumbs-up, come here, go away, okay, stop, and wave (Figure 2B). To evaluate the child’s understanding of the gestures and ensure their engagement, questions about the meaning were asked after each trial (i.e., “What were you/was I/were we doing?”, “What does it mean, when do you make that gesture?”). The accuracy of the child’s response was recorded to assess their gestural understanding. All children completed a total of 18 randomized trials, with 6 trials per condition, throughout the session (Figure 2C). Each trial consisted of a 10 s pre-stimulation, a 16 s stimulation, and a 16 s post-stimulation period. During the pre- and post-stimulation periods, participants were instructed to focus on a crosshair placed in front of them. These periods were included to account for baseline drifts in the fNIRS signal and to allow the hemodynamic response to return to baseline before commencing the next trial. Additionally, we used the postural praxis subtest of the Sensory Integration & Praxis Test (SIPT-PP; Ayres,1989) to assess the children’s praxis performance. During the SIPT-PP test, each child sat face-to-face with an adult tester and imitated the tester’s poses in a mirroring fashion, with a total of 17 poses. All testing sessions were video-recorded for future behavioral coding purposes.

### 2.3. fNIRS Data Collection

**Figure 3:**
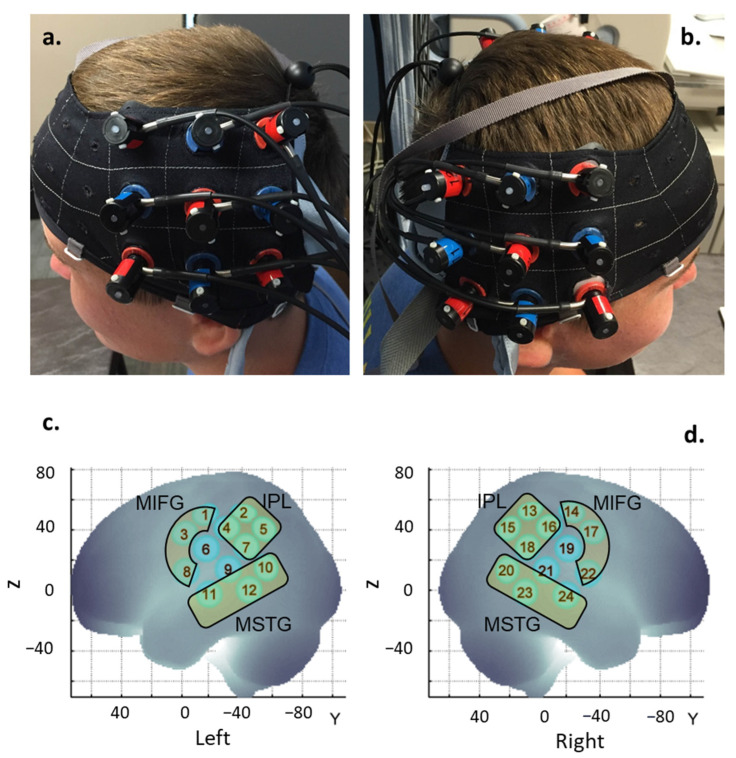
Probe placement (**a**,**b**) and spatial registration output (**c**,**d**). Written permission has been taken for the publication of participant pictures. MIFG = middle and inferior frontal gyrus, MSTG = middle and superior temporal gyrus; IPL = inferior parietal lobe. Numbers in **c** and **d** represent the channels in each hemisphere.

The Hitachi fNIRS system (ETG-4000, Hitachi Medical Systems, Inc., Tokyo, Japan) was employed to record the hemodynamic changes over the regions of interest (ROIs). Two 3 × 3 probe sets were positioned over the bilateral frontal, parietal, and temporal regions. Each probe set was aligned with the tragus point of the ear in the middle column, while the lowest row was placed just above the ear (T3 position of the International 10–20 system, Figure 3) [62]. Each fNIRS probe set consists of 5 infrared emitters and 4 receivers, arranged in an alternating pattern. The adjacent pair of probes (consisting of one emitter and one receiver) were spaced 3 cm apart. The emitter emitted two wavelengths of infrared light (695 and 830 nm) that passed through the skull, creating a banana-shaped arc and reaching the cortical area. The infrared light scattered around the tissues and was detected by the receiver. By employing the modified Beer–Lambert law, the attenuation of infrared light was used to calculate the changes in concentrations of oxygenated (HbO_2_) and deoxygenated hemoglobin (HHb) chromophores [63]. Increased HbO_2_ concentration and decreased HHb concentration are indicative of neural activation in the underlying cortical region when a brain region becomes active [63].

### 2.4. Spatial Registration Approach

During spatial registration, the 3D locations of the standard cranial landmarks (nasion, inion, left and right tragus points of the ears, and the Cz position of the International 10–20 system) were recorded, along with the position of each probe, using the motion tracking system of the ETG-4000 3D positioning unit. We used the anchor-based, spatial registration method developed by Tsuzuki et al. (2012) to transform the 3D location of each channel from the reference coordinate system to the Montreal Neurological Institute (MNI)’s coordinate system [64]. The channel positions (MNI coordinates) from all participants were averaged. Next, using the structural information from an anatomical database [64,65], we estimated the channel positions within a standardized 3D brain atlas and labeled them using the LONI Probabilistic Brain Atlas (LPBA) [50,51,52,53,54,55,56,57,58,59,60,61,62,66,67]. Based on the estimation, we assigned the 24 channels to three regions of interest (ROI) (Figure 3; Supplementary Table S1). The three ROIs comprised: (i) the middle and inferior frontal gyrus (MIFG; consists of channels 1, 3, and 8 on the left and channels 14, 17, and 22 on the right; Figure 3c,d); (ii) the middle and superior temporal gyrus (MSTG, consists of channels 10, 11, and 12 on the left and channels 20, 23, and 24 on the right; Figure 3c,d); (iii) inferior parietal lobe (IPL, consists of channels 2, 4, 5, and 7 in the left and channels 13, 15, 16, and 18 on the right, Figure 3c,d). Channels 6, 9, 19, and 21 have been excluded due to spatial uncertainty (Figure 3; Supplementary Table S1).

### 2.5. Video Coding for Postural Praxis and Communicative Gestures Tasks

Two student researchers coded the video to determine the number of spatial and temporal errors made by the children during the Do and Together conditions. To ensure reliability, 20% of the videos were coded, resulting in high inter- and intra-rater reliability (inter-rater reliability: 91%; intra-rater reliability: 99%). Spatial errors were identified when the child used joints/body parts that differed from those used by the social partner or depicted on the picture card (e.g., making a fist instead of showing the palm when waving hi), exhibited abnormal hand orientation (e.g., palm facing self when waving hi), or moved in the wrong direction (e.g., moving forward-backward instead of side to side when waving hi). Temporal errors were coded if the child’s movement was slower or faster compared to the tester. For the SIPT-PP test, the first author and a student researcher coded the video to identify modulation and spatial errors for each pose. Similarly, for this scheme, 20% of the videos were coded for reliability, and high inter- and intra-rater reliability was achieved (inter-rater reliability: 98%; intra-rater reliability: 99%). A spatial error was coded if the child used incorrect hand orientation or different joints or body parts compared to the tester. Modulation errors were coded when the child’s range of motion at joints was insufficient or exaggerated in comparison to the tester.

### 2.6. Processing Cortical Activation Data

**Figure 4:**
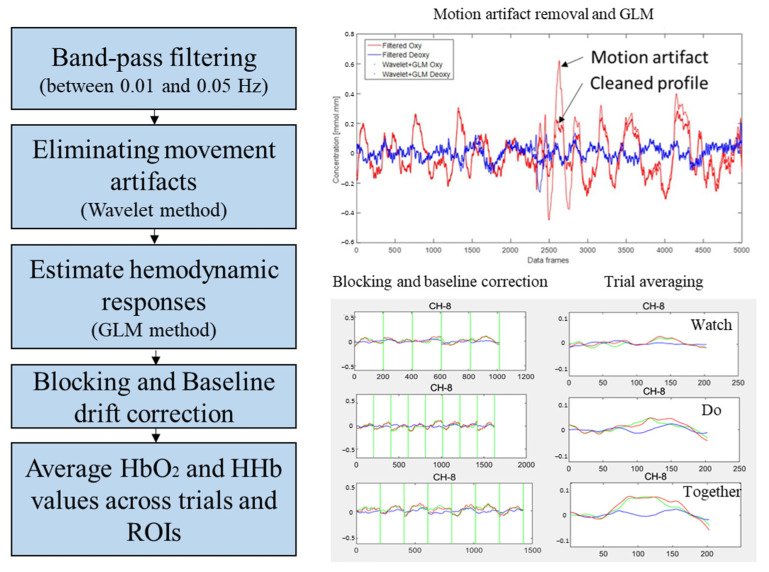
Data processing pipeline. Red lines indicate HbO_2_ concentration, blue lines indicate HHb concentration, while green lines indicate total concentration.

We utilized a custom MATLAB program (Version: 8.1.0.604 (R2013a)) that incorporates the open-source software Hitachi PoTATo (Version 3.8) [68] and Homer-2 for the analysis of fNIRS data [69]. Consistent with our previous papers [50,51,52,53], the following pre-processing steps were performed: (1) the data were band-pass filtered between 0.01 and 0.05 Hz to remove lower and higher frequency noises, including environment light artifacts; (2) motion artifacts were eliminated using the wavelet method [69,70]; (3) the hemodynamic response was estimated using the GLM method, including Gaussian basis functions and a 3rd order polynomial drift regression [69]; (4) baseline drifts were corrected by subtracting the linear trend between the pre- and post-stimulation baselines from values in the stimulation period [68]; (5) HbO_2_ values (similarly for HHb values) were averaged across the frames of the stimulation period for each trial; (6) data were averaged across channels within the same ROI based on our spatial registration output (see channel assignments in Supplemental Table S1). Figure 4 presents the data processing pipeline. We reported HbO_2_ profiles as they exhibit a greater range and signal-to-noise ratio compared to the HHb, and the HbO_2_ signals have been more frequently reported in the previous fNIRS literature [70].

### 2.7. Data Exclusion

A trained researcher conducted video coding to exclude trials in which the child did not follow task instructions or engaged in conversation with the partners during the stimulation period. For children with ASD, 3.5% of Watch trials, 2.1% of Do trials, and 2.1% of Together trials were excluded based on these criteria. In contrast, no trials were excluded for TD children in any of the conditions. Additionally, the first author screened the fNIRS data to exclude channels with no signals, likely due to bad probe-scalp contacts. In the children with ASD, 4.3% of the data in Watch trials, 2.1% in Do trials, and 3.0% in Together trials were excluded. For the TD children, 2.6% of the data in Watch trials, 3.1% in Do trials, and 2.7% in Together trials were excluded for this reason.

### 2.8. Statistical Analyses

For the analysis of postural praxis and communicative gesture performance, we used t-tests to assess group differences. In the case of cortical activation, we conducted repeated-measures ANOVA using IBM SPSS (SPSS, Inc. Chicago, IL, USA). The ANOVA included within-group factors of condition (Watch, Do, Together), hemisphere (left, right), and region of interest (MIFG, MSTG, IPL), as well as a between-group factor of group (TD, ASD). Since there were significant differences in IQ scores between TD and ASD groups, we included Full Scale IQ as a covariate. Greenhouse–Geisser corrections were applied when the data violated the sphericity assumption based on Mauchly’s test of sphericity. To address the issue of multiple comparisons, we used the false discovery rate (FDR) method for multiple post-hoc comparisons [71]. Specifically, the *p*-threshold was adjusted by multiplying 0.05 with the ratio of unadjusted *p*-value rank to the total # of comparisons (*p*-threshold for ith comparison = 0.05 × i/n; where n = total # of comparisons). The unadjusted *p*-values were ranked from low to high, and statistical significance was declared if the unadjusted *p*-value was lower than the adjusted *p*-value threshold. Lastly, Pearson correlations were conducted to examine correlations between cortical activation, SIPT, VABS communication and socialization subscales, ADOS, and SCQ scores.

## 3. Results

### 3.1. Postural Praxis and Communicative Gesture Performance

**Figure 5:**
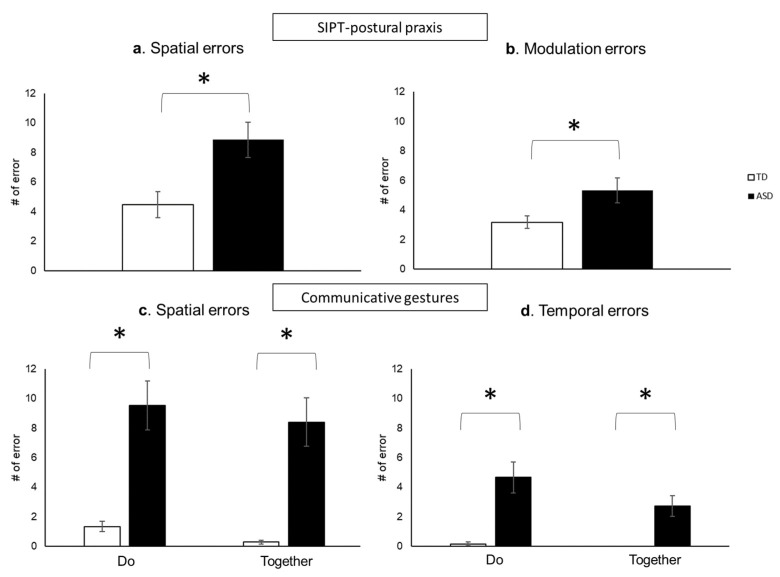
The spatial (**a**) and modulation errors (**b**) during the SIPT-PP test, as well as the spatial (**c**) and temporal errors (**d**)during Do and Together conditions of the communicative gesture tasks. * indicates significant difference between groups (*p* < 0.05).

Children with ASD showed more spatial and modulation errors during the postural praxis subtest of the SIPT-PP test compared to the TD children (*p*s < 0.05; Figure 5a,b). During the Do and Together conditions of the communicative gesture task, children with ASD showed significantly more spatial and temporal errors compared to the TD children (*p*s < 0.001, Figure 5c,d).

### 3.2. Cortical Activation during the Communicative Gesture Task

**Figure 6:**
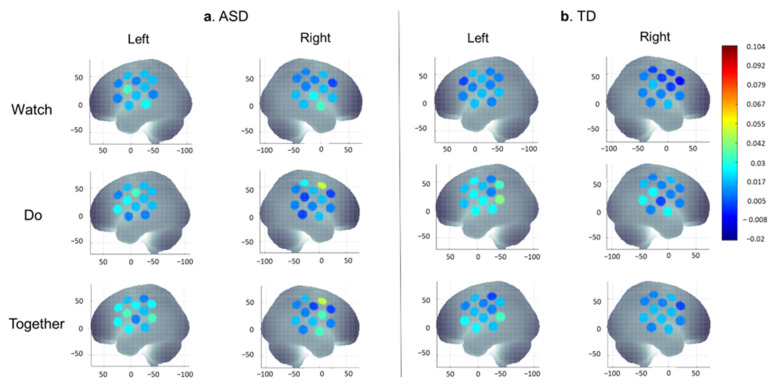
A visual representation of averaged HbO_2_ concentration during Watch, Do, and Together conditions in children with ASD (**a**) and TD children (**b**). HbO_2_ values on *Y*-axis range from 0 indicated by blue to 0.104 indicated by red and shades in between.

The Group × Condition × Hemisphere × Region four-way repeated ANOVA revealed significant main effects of Hemisphere (F (1.0, 211.0) = 9.6, *p* < 0.05) and Region (F (2.0, 422.0) = 3.6, *p* < 0.05), significant 2-way interactions of Group × Condition (F (2.0, 422.0) = 3.1, *p* < 0.05) and Group × Hemisphere (F (1.0, 211.0) = 6.1, *p* < 0.05), as well as significant/trend for 3-way interactions of Group × Condition x Region (F (4.0, 844.0) = 2.6, *p* < 0.05) and Group × Hemisphere × Region (F (2.0, 422.0) = 2.4, *p* = 0.09). The three-way interactions did not covary with the Full-Scale IQ score (*p*s > 0.05); therefore, post-hoc analyses were conducted to explore the interactions. The averaged HbO_2_ concentration during Watch, Do, and Together conditions in children with and without ASD is visually presented in Figure 6a,b. Detailed means and SEs of HbO_2_ concentration were presented in Supplementary Table S2, while the results of post-hoc analyses are presented in Supplementary Table S3.

#### 3.2.1. Group Differences

**Figure 7:**
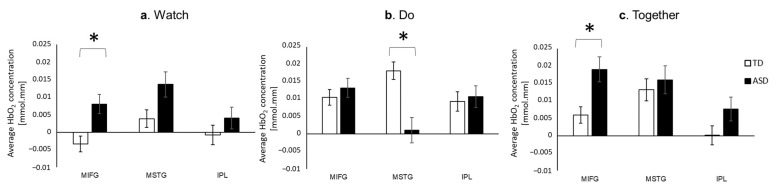
Group differences in average HbO_2_ concentration during Watch (**a**), Do (**b**), and Together (**c**) conditions. * indicates a significant difference between groups (*p* < 0.05).

During the Watch and Together conditions, children with ASD showed significantly higher MIFG, with a similar trend of greater IPL activation, compared to the TD children (MIFG, *p*s < 0.01, Figure 7a,c). In contrast, during the Do condition, the children with ASD showed significantly lower MSTG activation compared to the TD children (*p* < 0.01, Figure 7b).

#### 3.2.2. Condition-Related and Regional Differences

**Figure 8:**
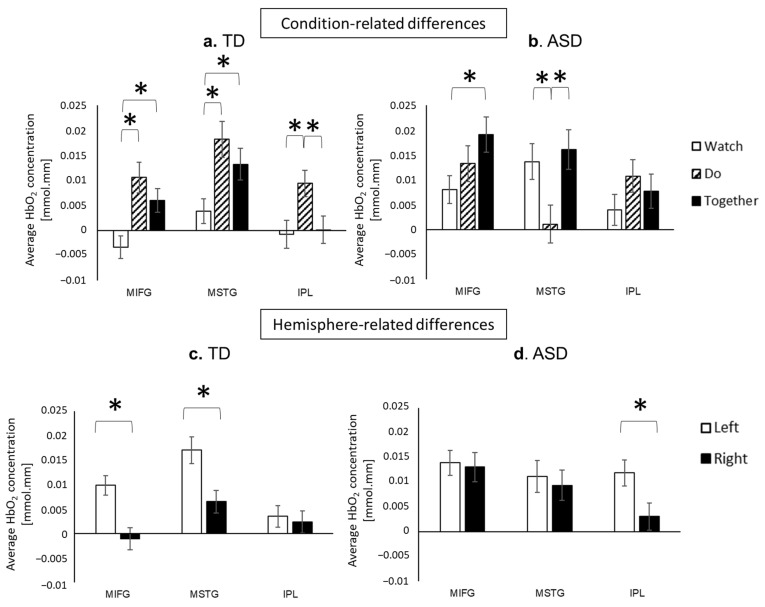
Conditional (**a**,**b**) and hemispheric differences (**c**,**d**) in average HbO_2_ concentration for TD children and children with ASD. * indicates significant difference between groups (*p* < 0.05).

TD children showed greater MIFG and MSTG activation during Do and Together conditions compared to the Watch condition (*p*s < 0.01, Figure 8). In terms of the IPL activation, TD children showed the highest activation during Do compared to the Watch and Together conditions (*p*s < 0.01, Figure 8). In contrast, the children with ASD showed greater MIFG activation during Together than the Watch condition (*p* < 0.01), and the lowest MSTG activation during Do compared to the Watch and Together conditions (*p*s < 0.05, Figure 8b). There were no significant condition-related differences in the IPL region among children with ASD (*p*s > 0.05, Figure 8b). Regarding regional differences, TD children showed the greatest activation in the MSTG, with significantly greater MSTG activation compared to MIFG during the Watch condition (*p* = 0.01) and greater MSTG activation compared to IPL during the Together condition (*p* < 0.01). On the contrary, children with ASD showed the lowest activation in the MSTG during the Do condition that was much lower than the MIFG and IPL regions (*p*s < 0.05). 

#### 3.2.3. Hemispheric Differences

Both TD children and children with ASD showed left-lateralized cortical activation during the communicative gesture tasks, albeit in different ROIs. Specifically, TD children showed left-lateralized (left > right) activation over the MIFG and MSTG regions (*p*s < 0.001, Figure 8c), while the children with ASD showed left-lateralized activation only in the IPL region (*p* < 0.01, Figure 8d). 

### 3.3. Correlation between Praxis, Communicative Gestures, and Cortical Activation

There were correlations between cortical activation and SIPT-PP performance in the TD children compared to the children with ASD (TD: 11 survived FDR corrections; ASD: only 1 survived FDR corrections). Furthermore, a greater number of correlations were found in the right compared to the left hemisphere (right: 10 survived FDR corrections; left: 2 survived FDR corrections). Specifically, in TD children, right MIFG and right IPL activation during Watch and Do conditions were correlated with the SIPT-PP spatial and modulation errors (r values ranging from 0.241 to 0.418, *p* < 0.02; Table 2). In addition, the TD children also showed correlations between left MIFG and left MSTG activation during Do condition and SIPT-PP modulation errors (r value = 0.322 and 0.270, *p*s < 0.01; Table 2). Both TD children and children with ASD showed significant correlations between right MIFG activation during the Together condition and the SIPT-PP modulation errors (r values = 0.279 and −0.314, *p*s < 0.01, Table 2). 

### 3.4. Correlation between VABS, ADOS, SCQ, and Cortical Activation in Children with and without ASD

TD children with higher VABS communication scores showed greater right MSTG activation during the Do condition (r = 0.334, *p* < 0.01). Similarly, children with ASD showed a similar relationship between VABS communication scores and MSTG activation; however, these correlations did not survive FDR corrections (Table 3). In addition, the activation over MSTG during the Do condition is correlated with the SCQ scores in children with ASD (r = −0.388, *p* < 0.001, Table 4). Although there were multiple meaningful correlations between cortical activation and ADOS scores in multiple regions, none of them survived FDR corrections (Table 4).

## 4. Discussion

Children with ASD have difficulties in perceiving, producing, and imitating communicative gestures, which can hinder their social connections and have cascading negative effects on language and social-cognitive development [3,4,44]. Previous fMRI studies have reported atypical prefrontal activation during gesture observation and abnormal fronto-parietal activation during gesture production/imitation in children with ASD [9,10]. However, the findings were limited to video observations or copying of hand movements from picture cards, lacking real-world interactive contexts. To overcome this limitation, the current study used fNIRS to investigate the neural activity during naturalistic, face-to-face interactions involving gesture observation, production, and imitation in children with and without ASD. Consistent with previous behavioral findings, children with ASD exhibited impaired praxis and communicative gesture performance, exhibiting more modulation and spatial errors during the praxis test (assessed by SIPT-PP) as well as more spatial and temporal errors when producing and imitating communicative gestures. Furthermore, compared to their TD peers, children with ASD showed hyperactivation in the MIFG and IPL regions during gesture observation and imitation, while showing hypoactivation in the MSTG regions during gesture production. For condition-related differences, TD children exhibited greater MIFG and MSTG activation during the movement-related conditions (Do and Together) compared to action observation (Watch), along with greater IPL activation during Do compared to Watch and Together conditions. Conversely, children with ASD showed notably low MSTG activation during Do compared to Watch and Together conditions. Regarding hemispheric differences, TD children showed left-lateralized activation over the MIFG and MSTG regions, whereas children with ASD recruited left-lateralized IPL regions. Lastly, for regional differences, TD children showed greatest activation in the MSTG, with greater MSTG compared to MIFG activation during the Watch condition and greater MSTG compared to IPL activation during the Together condition. In contrast, children with ASD showed the lowest activation in the MSTG during the Do condition when compared to MIFG and IPL regions. In short, children with ASD recruited distinct neural networks that differed from their TD peers during gestural tasks. 

### 4.1. Impaired Praxis and Communicative Gesture Performance in Children with ASD

Our behavioral findings from the postural praxis and communicative gesture tasks provide further evidence of atypical gesture production and imitation in children with ASD, as indicated by increased spatial, modulation, and temporal errors compared to their TD peers. These findings align with previous studies involving various hand gestures and upper-limb movements [3,4,9,44]. For example, using a charades game paradigm, Fourie et al. (2020) found poor gestural performance quality and atypical hand use in children with ASD [9]. Moreover, in the context of gestural communication (e.g., fingerspelling), deaf children with ASD exhibited greater errors in pace, sequence precision, accuracy, and body part use, along with longer processing time compared to deaf children without ASD [3]. The increased spatial and modulation errors in children with ASD may be attributed to their atypical visuospatial processing, insufficient internal action models, limited movement repertoire of hand gestures, and motor incoordination [33,34,35,36,72,73]. In addition to the spatial components, children with ASD also demonstrate atypical timing when producing and imitating gestures [74]. In our experimental paradigm, we instructed the children to perform gestures at a “comfortable speed” (Do condition) or imitate the tester’s hand movements in a rhythmic manner (Together condition), which involves both internal timing and interpersonal synchrony. Regarding the internal timing of spontaneous gesture production, a previous study found mismatched timing of speech and gestures in children with ASD during a narrative task [75]. These children exhibited more variable and different speeds in their gestural movements compared to the standard timing of the test, indicating difficulties with internal timing for gesture production (i.e., more temporal errors). During interpersonal synchrony tasks, our team has identified poor synchrony skills in children with ASD during joint reaching and swaying tasks [45,47,50,51]. Interpersonal synchrony requires perceiving cues from one’s partner, anticipatory control, and reactive adjustments to match their partner’s actions [76,77]. Children with ASD have difficulties in social monitoring, sensorimotor integration, and impaired executive functions, such as cognitive flexibility, planning, inhibition, and working memory, which may impact their ability to imitate and synchronize their communicative gestures with those of their partners [78,79,80].

### 4.2. ASD-Related Regional Hyperactivation in the MIFG and IPL during Gesture Observation and Imitation

In terms of cortical activation, we observed greater MIFG and IPL activation in children with ASD compared to TD children during gesture observation and imitation. Additionally, children with ASD showed greater activation in the MIFG and IPL regions compared to MSTG activation. MIFG and IPL activation during gesture imitation was associated with modulation errors during the praxis task (SIPT-PP) in children with ASD, suggesting potential difficulties in gesture understanding/interpretation or action planning. The MIFG and IPL are part of the frontoparietal network associated with reasoning and physical judgment [17,18]. Specifically, being part of the gesture observation network, the IFG region is involved in understanding the semantic meaning and emotional aspects of gestures [9,22,40,41]. The observed hyperactivation over the IFG region might suggest that more effort is needed for children with ASD to process/interpret the semantic and emotional components of the observed gestures. This neural finding aligns with the behavioral study reporting reduced pupillary dilation (indicating increased mental effort) in children with ASD during gesture observation [30]. The greater activation of the IPL compared to STS in children with ASD may also indicate that they perceive social gestures as abstract actions that are more represented in the IPL vs. STS [81]. In terms of gesture production/imitation, the MIFG region is important in attention selection and responses during imitation [82,83]. Moreover, as part of the OEMS, IFG plays an important role in movement imitation/synchrony [20]. The frontoparietal network (MIFG and IPL) is important for motor/action planning of gestures and other complex actions [84,85], and the observed hyperactivation in children with ASD may suggest difficulties with action planning of gestures. Overall, the atypical activation observed in the MIFG and IPL regions may indicate an atypical and/or a potentially compensatory mechanism wherein children with ASD rely more on reasoning and physical understanding to compensate for their difficulties in grasping the social–emotional aspects underlying gestures [17,18]. 

### 4.3. ASD-Related Relative Regional Hypoactivation over STS during Gesture Production

During gesture production (Do condition), children with ASD exhibited reduced MSTG activation, and the level of activation in MSTG was correlated with their ASD symptoms as measured by SCQ and ADOS. STS, along with other temporal regions, is important for establishing visuomotor correspondence by comparing observed actions with one’s own internal movement repertoire [26,42]. It is also involved in providing feedback control of self-generated movements [86]. The decreased activation in MSTG during gestural production may indicate an atypical internal movement repertoire and impaired feedback control of movement in children with ASD. Moreover, STS, along with the other temporal regions, is part of the social brain network [87]. A previous study by Joue et al. (2020) found greater involvement of the temporal lobe during the imitation of communicative gestures compared to tool-use gestures, highlighting its importance in social information processing [29]. Studies using fNIRS and EEG have also observed increased activation in STS during natural social interaction and when individuals are being observed [87,88]. Children with ASD might have difficulties effectively interpreting social gestures, perceiving them as abstract actions rather than social cues, which can affect their planning and production of communicative gestures. It is worth noting that our previous publications using similar observation, action, and imitation conditions during various social actions, including reaching, body sway, and a socially cooperative building task, have also shown similar patterns of hyper- and hypoactivation [51,52,53].

### 4.4. Left-Lateralized Neural Networks for Communicative Gestures in Children with and without ASD

Similar to language networks, communicative gesture tasks engage a predominantly left-lateralized cortical network [16]. In the current communicative gesture task, we specifically instructed the children to use their right hand to perform and imitate the communicative gestures, leading to more activation over the contralateral than the ipsilateral hemisphere (left lateralization). Although both groups of children exhibited left-lateralized cortical activation, the specific regions showing the lateralization differed between children with and without ASD. Specifically, TD children showed left lateralization in MIFG and MSTG regions, while the children with ASD showed left lateralization in the IPL region. This atypical regional lateralization suggests that children with ASD might recruit different neural circuits when performing and imitating communicative gestures. In fact, children with ASD are known to have altered connectivity patterns, including increased short-range connectivity within frontal, parietal, and temporal cortices, as well as reduced long-range connectivity between the fronto-parietal and fronto-temporal cortex [89].

### 4.5. Neurobiomarkers and their Clinical Implications in Gesture-Based Interventions

The current study extends our understanding of neurobiomarkers associated with ASD to naturalistic gestural communication and offers potential intervention strategies based on our cortical activation findings. Specifically, children with ASD showed hyperactivation over the MIFG and IPL regions compared to STS during observation and imitation of communicative gestures, suggesting increased efforts in perceiving, planning, and matching one’s own gestures to those of others. Children with ASD’s hypoactivation in the MSTG during gesture production suggests atypical internal movement repertoires and altered representations in social and abstract action planning networks [26,81,87]. These ASD-related neurobiomarkers have the potential to be used in the early identification of gestural difficulties in young children with ASD and could serve as objective measures for evaluating intervention efficacy [90,91]. On the other hand, the findings of the current study highlight the importance of movement in gestural communication. In the TD group, we observed greater cortical activation in MIFG and MSTG during conditions involving actions (Do and Together conditions) compared to gesture observation (Watch condition). Children with ASD also showed notably lower MSTG activation during gesture production compared to gesture observation and imitation. These findings suggest that relying solely on visual cues from picture cards might not be sufficient to engage internal representations and activate the social/abstract action networks in children with ASD. Conversely, imitation appears to be a valuable strategy for enhancing communicative gesture performance in children with ASD, as it elicited the highest activation over the MIFG and MSTG regions.

### 4.6. Limitations and Future Research Directions

Despite the promising findings, there are several limitations in our study that could be addressed in future research. Firstly, our use of the 24-channel probe set limited the coverage of the whole brain, potentially constraining our ability to capture comprehensive cortical activation patterns. Additionally, the validity and reliability of fNIRS as compared to other neuroimaging techniques are still not fully understood. Future studies should consider using full brain coverage and incorporating simultaneous recordings of fNIRS with other neuroimaging tools (e.g., EEG) to validate our findings [92]. Another limitation of our study was the relatively small sample size, which restricted our ability to explore potential behavioral and neural subgroups within the ASD population. Future studies with larger sample sizes could address this limitation and provide a more nuanced understanding of the heterogeneity within ASD. Lastly, the current fNIRS task focused on simple communicative gestures, which may not fully capture the complexity of naturalistic exchanges. To address this limitation, our research group is currently conducting a study involving a naturalistic charade game that includes more complex communicative gestures, facilitating a more ecologically valid assessment. In this ongoing study, a whole-brain probe set with enhanced prefrontal coverage is being utilized to better capture cortical activation during more complex, naturalistic interactions. 

## 5. Conclusions

The current study is the first to investigate the ASD-related differences in neural activity during naturalistic exchanges of communicative gestures. Compared to their TD peers, children with ASD exhibited hyperactivation in MIFG and IPL during observation and imitation, and hypoactivation over MSTG during gesture production. More importantly, greater activation in MSTG was observed when children with ASD imitated the gestures of others, as opposed to producing gestures solely based on visual cues from picture cards, highlighting the importance of active learning when engaging in communicative gestures. Furthermore, our study suggests potential neural biomarkers that could aid in understanding the mechanisms of gestural difficulties of children with ASD, as well as help monitor the effects of interventions that target gestural communication.

## Figures and Tables

**Table 1:** Demographic and related information for ASD and TD groups.

Characteristics	ASD Group (*n* = 15) Mean ± SE	TD Group (*n* = 17) Mean ± SE
Age	11.1 ± 0.9	10.8 ± 0.7
Sex	11 M, 4 F	11 M, 6 F
Race	10 C, 3 A, 2 MR	13 C; 1 A; 1 AI; 2 MR
Ethnicity	15 NH, 0 H	17 NH, 0 H
SES-Child	65.9 ± 5.1	69.7 ± 4.4
Handedness Coren’s score	Right-handed 33.5 ± 1.5	Right-handed 33.4 ± 1.8
SCQ	24.5 ± 3.2	-
ADOS Social affect Repetitive behavior	18.9 ± 1.9 13.7 ± 1.4 5.2 ± 0.6	-
Stanford-Binet IQ Full Scale IQ Verbal IQ Non-verbal IQ	82.1 ± 7.6 * 87.2 ± 7.9 * 71.23± 6.4 *	114.2 ± 1.7 114.6 ± 2.3 114.5 ± 1.7
VABS-II (SS) Communication (SS) Daily living (SS) Socialization (SS)	71.1 ± 3.2 * 73.5 ± 3.3 * 68.0 ± 3.8 * 76.6 ± 3.8 *	110.3 ± 2.9 109.2 ± 2.6 108.1 ± 3.4 110.5 ± 3.2
SRS (T scores)	78.7 ± 2.2 *	45.1 ± 1.3
ICS	4.2 ± 0.2 *	5.1 ± 0.2

SES-Child = Hollingshead Four-Factor Index of Socioeconomic Status; SCQ = Social Communication Questionnaire; ADOS = Autism Diagnostic Observation Schedule—2nd Edition; IQ = Intelligence Quotient; VABS-II = Vineland Adaptive Behavior Scale—2nd Edition; SS = Standard Score; SRS = Social Responsiveness Scale; ICS = Interpersonal Communication Scale; M = Male, F = Female; C = Caucasian, A = Asian, MR = Mixed-Race, AI = American Indian. H = Hispanic; NH: Non-Hispanic; * indicates significant differences between children with and without ASD.

**Table 2:** The correlations between cortical activation and SIPT-PP and COM performance.

r-Values	SIPT-PP Spatial Error			SIPT-PP Modulation Error		
	W	D	T	W	D	T
TD						
Left hemisphere						
MIFG MSTG IPL	0.031 0.072 0.209*	0.113 0.181 0.125	−0.054 −0.014 −0.148	−0.082 0.135 0.056	**0.322** ** 0.270 ** 0.063	0.187 0.116 −0.167
Right hemisphere						
MIFG MSTG IPL	**0.329** ** 0.046 **0.286** **	**0.323** ** 0.123 0.341 **	0.159 0.066 0.132	**0.249** ** −0.018 **0.241** *	**0.418** ** 0.026 **0.294** **	**0.279** ** 0.035 0.009
ASD						
Left hemisphere						
MIFG MSTG IPL	−0.083 −0.013 0.031	−0.108 −0.100 −0.037	−0.185 −0.047 0.023	0.000 −0.004 0.162	−0.171 −0.153 −0.086	−0.157 0.026 −0.108
Right hemisphere						
MIFG MSTG IPL	−0.177 0.054 0.169	−0.041 0.082 0.085	**−0.294** ** −0.059 0.003	−0.192 0.078 0.066	−0.138 −0.137 −0.094	**−0.314** ** −0.088 −0.182

r values of correlations between activation and errors are shown. Bold font and * indicate *p* < 0.05; Bold font and ** indicate *p* < 0.01. Shaded values indicate *p* values survived FDR corrections.

**Table 3:** The correlations between cortical activation and VABS communication and socialization scores.

r-Values	VABS-Communication			VABS-Socialization		
	W	D	T	W	D	T
TD						
Left hemisphere						
MIFG MSTG IPL	0.024 0.009 0.087	0.107 0.134 0.152	0.162 **0.237** * -0.274	−0.094 −0.110 0.006	−0.112 −0.103 0.063	**−0.247** ** 0.138 −0.133
Right hemisphere						
MIFG MSTG IPL	0.047 0.049 0.045	0.035 **0.334** ** 0.151	−0.089 **0.209** * −0.103	−0.103 −0.113 −0.104	−0.069 **0.214** * 0.013	−0.024 **0.211** * −0.081
ASD						
Left hemisphere						
MIFG MSTG IPL	−0.064 0.081 −0.026	−0.064 0.120 −0.139	−0.095 0.088 −0.172	−0.177 0.034 −0.111	0.130 0.132 −0.111	**−0.266** ** 0.000 −0.170
Right hemisphere						
MIFG MSTG IPL	0.057 **0.273** ** −0.027	0.052 **0.205** * 0.161	−0.009 **0.194** * −0.083	0.004 **0.223** * −0.003	0.089 0.186 0.147	−0.018 0.162 −0.062

r values of correlations between activation and VABS scores are shown. Bold font and * indicate *p* < 0.05; Bold font and ** indicate *p* < 0.01. Shaded values indicate *p* values survived FDR corrections.

**Table 4:** The correlations between cortical activation, ADOS, and SCQ scores.

r-Values	ADOS-SA			ADOS-RRB			SCQ		
	W	D	T	W	D	T	W	D	T
ASD									
Left hemisphere									
MIFG MSTG IPL	0.138 0.126 **0.241** *	−0.087 −0.077 −0.052	0.011 0.188 0.086	0.033 0.013 0.190	**−0.299** * −0.305 * −0.011	−0.130 0.003 0.054	0.093 0.019 0.142	**−0.243** * −0.388 ** −0.081	0.071 0.020 0.077
Right hemisphere									
MIFG MSTG IPL	−0.019 −0.067 0.137	−0.035 −0.161 **−0.248** *	−0.048 **−0.247** * −0.024	−0.102 0.085 0.104	**−0.242** * −0.004 −0.051	−0.186 −0.017 0.070	−0.006 −0.113 0.165	**−0.250** * −0.109 −0.166	−0.047 −0.106 **0.220** *

r values of correlations between activation and ADOS and SCQ scores are shown. Bold font and * indicate *p* < 0.05; Bold font and ** indicate *p* < 0.01. Shaded values indicate *p* values survived FDR corrections.

## Data Availability

The data presented in this study are available on request from the corresponding author. The data are not publicly available due to restrictions associated with participants’ privacy.

## Supplementary Materials

The following supporting information can be downloaded at: https://www.mdpi.com/article/10.3390/brainsci13091284/s1, Table S1: Channel assignments based on the anchor registration approach title; Table S2: Means and standard errors of HbO_2_ concentration in children with and without ASD during Watch, Do, and Together conditions of the communicative gesture task; Table S3: The post-hoc analyses for 3-way interactions of Group × Condition × Region and Group × Hemisphere × Region.
