# Supplementary material for: Autism-Related Differences in Cortical Activation When Observing, Producing, and Imitating Communicative Gestures: An fNIRS Study
Source: Brain Sci. 2023 Sep 4;13(9):1284. doi: 10.3390/brainsci13091284 (PMC10527424; doi:10.3390/brainsci13091284)
Supplement: Supplementary file 1 [file brainsci-13-01284-s001.zip › brainsci-2477265-supplementary.pdf]

ASD-Related Differences in Cortical Activation When Observing, Producing, and Imitating Communicative Gestures: An fNIRS Study

Su, W.,[1,2] Culotta, M.,[1,2] Mueller, J.,[3] Tsuzuki, D.,[4] Bhat, A. N.[1,2,5]

Supplementary Table S1. Channel assignments based on the anchor registration approach. For each channel, the spatial location in the MNI coordinate system and the probability of covering different brain regions are shown. The channels are symmetrically divided across the two hemispheres (left, right). The color-coded channels were considered within a specific ROI (MIFG, IPL, and MSTG).

Side	Ch	MNI's coordinate system			MIFG regions			IPL regions			MSTG regions		Assigned region
		X	Y	Z	Inferior frontal gyrus	Middle frontal gyrus	Precentral gyrus	Postcentral gyrus	Supramarginal gyrus	Angular gyrus	Middle temporal gyrus	Superior temporal gyrus	
**left**	1	-50.00	6.00	51.67	-	47.0	53.0	-	-	-	-	-	MIFG
	2	-57.33	-24.67	54.67	-	-	-	52.4	47.6	-	-	-	IPL
	3	-52.00	23.33	38.33	0.022	97.8	-	-	-	-	-	-	MIFG
	4	-61.67	-9.33	42.33	-	-	24.6	72.0	3.5	-	-	-	IPL
	5	-64.00	-39.00	44.67	-	-	-	-	92.9	7.1	-	-	IPL
	6	-62.67	7.33	27.67	9.3	7.2	83.4	-	-	-	-	-	Excluded**
	7	-68.00	-24.33	31.67	-	-	-	5.0	94.0	-	-	0.9	IPL
	8	-59.00	24.33	11.33	100.0	-	-	-	-	-	-	-	MIFG
	9	-68.00	-9.33	16.33	-	-	1.6	84.2	2.8	-	-	11.4	Excluded*
	10	-69.00	-39.67	18.33	-	-	-	-	25.8	-	-	74.2	MSTG
	11	-62.33	3.67	-1.67	4.0	-	20.5	-	-	-	0.7	74.7	MSTG
	12	-71.00	-26.67	1.33	-	-	-	-	-	-	62.3	37.7	MSTG
**right**	13	56.67	-27.00	57.00	-	-	-	5.1	94.9	-	-	-	IPL
	14	52.00	5.00	52.67	-	27.3	71.5	1.2	-	-	-	-	MIFG
	15	64.00	-42.33	46.33	-	-	-	-	54.3	45.7	-	-	IPL
	16	64.00	-10.67	43.33	-	-	3.2	45.9	50.9	-	-	-	IPL
	17	54.33	21.33	37.67	17.3	60.1	22.7	-	-	-	-	-	MIFG
	18	70.00	-26.33	31.67	-	-	-	2.5	95.1	2.5	-	-	IPL
	19	66.00	5.33	27.33	-	-	64.3	35.7	-	-	-	-	Excluded*
	20	70.00	-44.33	16.67	-	-	-	-	5.7	6.1	35.4	52.9	MSTG
	21	70.00	-12.33	14.67	-	-	-	48.6	1.9	-	-	49.5	Excluded*
	22	62.00	21.33	11.33	51.1	-	48.9	-	-	-	-	1.6	MIFG
	23	73.00	-30.33	-0.33	-	-	-	-	-	-	71.1	28.9	MSTG
	24	67.00	0.33	-3.67	-	-	-	-	-	-	19.7	80.3	MSTG

Supplementary Table S2. Means and standard errors of HbO_2 concentration in children with and without ASD during Watch, Do, and Together conditions of the communicative gesture task.

**Group activation data**	**Watch**		**Do**		**Together**	
	**Mean**	**SE**	**Mean**	**SE**	**Mean**	**SE**
**TD**						
Left hemisphere						
MIFG	0.002	0.003	0.016	0.004	0.011	0.003
MSTG	0.006	0.004	0.026	0.005	0.019	0.005
IPL	0.002	0.004	0.010	0.004	-0.001	0.004
Right hemisphere						
MIFG	-0.009	0.003	0.005	0.004	0.001	0.004
MSTG	0.001	0.003	0.010	0.005	0.008	0.004
IPL	-0.003	0.004	0.008	0.004	0.002	0.004
**ASD**						
Left hemisphere						
MIFG	0.011	0.004	0.012	0.005	0.019	0.004
MSTG	0.010	0.005	0.004	0.006	0.019	0.006
IPL	0.008	0.004	0.014	0.004	0.014	0.005
Right hemisphere						
MIFG	0.005	0.004	0.015	0.005	0.019	0.006
MSTG	0.017	0.005	-0.002	0.005	0.013	0.005
IPL	0.000	0.004	0.007	0.005	0.002	0.005

Supplementary Table S3. The post-hoc analyses for 3-way interactions of Group x Condition x Region and Group x Hemisphere x Region.

**Comparison**	**Significant *p* values**	**Direction of effect**
**Group related difference**		
Group x Condition x Region		
Watch, MIFG	0.001	ASD > TD [a]
Watch, MSTG	0.026	ASD > TD [b]
Do, MSTG	0.001	TD > ASD [a]
Together, MIFG	0.002	ASD > TD [a]
Group x Hemisphere x Region		
Left, IPL	0.015	ASD > TD [a]
Right, MIFG	<0.001	ASD > TD [a]
**Condition related difference**		
Group x Condition x Region		
TD, MIFG	<0.001	Do > Watch [a]
	0.001	Together > Watch [a]
TD, MSTG	0.001	Do > Watch [a]
	0.019	Together > Watch [a]
TD, IPL	0.002	Do > Watch [a]
	0.010	Do > Together [a]
ASD, MIFG	0.007	Together > Watch [a]
ASD, MSTG	0.011	Watch > Do [a]
	0.002	Together > Do [a]
**Hemisphere related difference**		
Group x Hemisphere x Region		
TD, MIFG	<0.001	Left > Right [a]
TD, MSTG	<0.001	Left > Right [a]
ASD, IPL	0.003	Left > Right [a]

a. The *p* values survived FDR correction
b. The *p* values < 0.05, but not survived FDR correction
